# Efficacy and safety of PI3K inhibitors combined with fulvestrant for HR+/HER2− advanced breast cancer: a systematic review and meta-analysis

**DOI:** 10.3389/fonc.2025.1556978

**Published:** 2025-06-04

**Authors:** Xuefeng Li, Hongxian Wang, Shuhui Lin, Tianwen Chen

**Affiliations:** ^1^ Day Surgery Care Unit, Shenzhen Nanshan People’s Hospital, Shenzhen, Guangdong, China; ^2^ Department of Breast and Thyroid Surgery, Shenzhen Nanshan People’s Hospital, Shenzhen, Guangdong, China; ^3^ Department of Radiation Oncology, Shenzhen Nanshan People’s Hospital, Shenzhen, Guangdong, China

**Keywords:** PI3K inhibitors, advanced breast cancer, progression-free survival, PIK3CA mutations, meta-analysis

## Abstract

**Objectives:**

This systematic review and meta-analysis aimed to evaluate the efficacy and safety of combination of Phosphatidylinositol 3-kinase (PI3K) inhibitors and fulvestrant in patients with advanced breast cancer (ABC) who are hormone receptor-positive (HR+) and human epidermal growth factor receptor 2-negative (HER2-).

**Methods:**

A systematic search was conducted across the PubMed, Cochrane Library, EMBASE databases and major conference websites (ASCO, ESMO, SABCS) to identify randomized controlled trials (RCTs) evaluating the combination of PI3K inhibitors and fulvestrant in the treatment of advanced breast cancer. Two independent reviewers systematically screened the literature, extracted data, and assessed the risk of bias for the included studies based on predefined criteria. Meta-analysis was subsequently performed using R software in accordance with the PRISMA guidelines.

**Results:**

A total of five randomized controlled trials (RCTs) involving 3,011 patients were included. The findings indicated that the combination of PI3K inhibitors and fulvestrant significantly improved progression-free survival (PFS) (HR = 0.74, 95% CI 0.67-0.80, P < 0.0001) and the objective response rate (ORR) (RR = 1.80, 95% CI: 1.39-2.35, P < 0.0001) compared to placebo plus fulvestrant. However, there was no statistically significant difference in clinical benefit rate (CBR) (RR = 1.10, 95% CI: 0.97-1.25, P = 0.1341). Subgroup analysis indicated that the predefined subgroup of PFS based on PIK3CA mutation status assessed by ctDNA was statistically significant (P = 0.0039), whereas the predefined subgroup of PFS based on PIK3CA mutation status assessed by tumor tissue was not statistically significant (P = 0.1514). In terms of adverse events, the incidence of grade ≥3 events was significantly increased in the PI3K inhibitors combined with fulvestrant group (RR=2.11, 95% CI: 1.73-2.58, P<0.0001), particularly hyperglycemia, rash, and transaminitis (ALT).

**Conclusion:**

The combination of PI3K inhibitors and fulvestrant significantly improved PFS and ORR in patients with advanced breast cancer. However, substantial dose-limiting toxicities associated with this therapeutic regimen restrict its broader clinical application. In patients with PIK3CA mutations detected on ctDNA analysis, PFS was significantly improved compared to those with wild-type PIK3CA, suggesting that ctDNA-based PIK3CA mutation status may serve as a potential biomarker for treatment response.

**Systematic review registration:**

PROSPERO, identifier CRD42023407466.

## 1 Introduction

Breast cancer is the most common malignant tumor among women worldwide and remains a leading cause of cancer-related mortality (1). Hormone receptor-positive, human epidermal growth factor receptor 2-negative (HR+/HER2−) breast cancer, which accounts for approximately 70% of all cases, is the most prevalent subtype (2–4). Over the past decade, significant advances in evidence-based medicine have fundamentally transformed the therapeutic landscape for hormone receptor-positive, HER2-negative (HR+/HER2-) advanced breast cancer. The combination of CDK4/6 inhibitors with endocrine therapy has emerged as the standard treatment, effectively delaying disease progression and improving patient outcomes (5–7). Nevertheless, advanced breast cancer remains incurable, as nearly all patients eventually develop therapeutic resistance and experience disease progression (8, 9). Consequently, the pursuit of novel therapeutic strategies to improve patient outcomes has become a critical area of research.

The phosphatidylinositol-3-kinase (PI3K) signaling pathway is extensively involved in critical cellular physiological functions and processes, including cell growth, proliferation, motility, and metabolism, playing a crucial role in breast cancer development and progression (10, 11). The PIK3CA gene, which encodes the PI3K catalytic subunit α isoform (p110α), is mutated in approximately 40% of HR+/HER2- advanced breast cancer patients (12–14). Substantial evidence indicates that PIK3CA mutations lead to aberrant activation of the PI3K/AKT/mTOR signaling pathway, contributing to endocrine therapy resistance in breast cancer and closely correlating with poor prognosis (15–17). Therefore, the combination of PI3K inhibitors with endocrine therapy has emerged as a promising therapeutic strategy to enhance treatment outcomes in patients with advanced breast cancer. The phase III SOLAR-1 trial demonstrated that the PI3K inhibitors Alpelisib significantly prolonged median PFS compared to the control group (18). Based on these findings, Alpelisib received approval from the U.S. Food and Drug Administration (FDA), becoming the first PI3K inhibitor approved for advanced breast cancer. However, other clinical trials have not demonstrated a significant PFS benefit (19). Furthermore, the notable dose-limiting toxicities associated with PI3K inhibitors warrant safety assessment of the combination regimen in expanded clinical trials. Therefore, we conducted this systematic review and meta-analysis to comprehensively assess the efficacy and safety of PI3K inhibitors combined with fulvestrant in advanced breast cancer, aiming to provide more reliable evidence-based recommendations for clinical practice.

## 2 Materials and methods

### 2.1 Literature search

This systematic review and meta-analysis was conducted in accordance with the preferred reporting items for systematic reviews and meta-Analyses (PRISMA) guidelines and was registered on PROSPERO (Registration No. CRD42023407466). To identify all eligible records, a comprehensive literature search was performed in the PubMed, Cochrane Library, and EMBASE databases, as well as in the conference websites of the American Society of Clinical Oncology (ASCO), the European Society for Medical Oncology (ESMO), and the San Antonio Breast Cancer Symposium (SABCS). Literature search was conducted up to December 16, 2024, without start date restrictions.

### 2.2 Inclusion and exclusion criteria

**Inclusion criteria**: This analysis included phase II and III randomized controlled trials (RCTs) that involved patients with histologically or cytologically confirmed HR+/HER2- advanced breast cancer, including both postmenopausal female and male patients. The advanced breast cancer is defined as either metastatic breast cancer or inoperable locally advanced breast cancer. In this study, the experimental group received a PI3K inhibitor plus fulvestrant, while the control group received a placebo plus fulvestrant. The primary endpoint was progression-free survival (PFS), while secondary endpoints included objective response rate (ORR), clinical benefit rate (CBR), and grade ≥3 adverse events.

**Exclusion criteria**: Single-arm trials, case-control studies, animal experiments, reviews, and studies not classified as Phase II or III randomized controlled trials were excluded. Studies were excluded if they lacked PI3K mutation status testing or included additional medications in either treatment arm. Non-English publications and studies with incomplete or missing crucial data were excluded. For duplicate publications of the same trial, only the most recent and comprehensive version was included. Ongoing or unpublished studies were excluded as well.

### 2.3 Outcome measures

The primary endpoint of the study was progression-free survival. Secondary endpoints included the objective response rate (ORR), which was assessed according to RECIST version 1.1 and defined as either a complete or partial response. Additionally, the clinical benefit rate (CBR) was evaluated, defined as complete response, partial response, or stable disease within 24 weeks. This study also monitored the incidence of grade ≥3 adverse events.

### 2.4 Study selection and data extraction

Two investigators (Xuefeng Li and Hongxian Wang) independently extracted data, resolving discrepancies through consensus. From each eligible study, the following information was collected: study name, design, treatment regimen, population characteristics, number of participants, progression-free survival (PFS), objective response rate (ORR), clinical benefit rate (CBR), and the incidence of grade ≥3 adverse events. The risk of bias in the included studies was assessed using the Cochrane Collaboration’s tool, consisting of six domains: random sequence generation, allocation concealment, blinding of participants and personnel, blinding of outcome assessment, incomplete outcome data, and selective reporting. Two investigators independently evaluated the risk of bias in each study. Any disagreements were resolved through discussion between reviewers, with arbitration by a third investigator when necessary.

### 2.5 Statistical analysis

Statistical analysis were performed using R software (version 4.4.1). Pooled hazard ratio (HR) and relative risk (RR) was calculated using both Mantel-Haenszel (M-H) and inverse variance (I-V) methods, with studies weighted according to the generic inverse variance approach. Based on heterogeneity assessment, either a fixed-effects or a random-effects model was applied to compute two-sided 95% confidence intervals (CIs). Heterogeneity between studies was assessed using Cochran’s Q test (χ²) and quantified by I² statistics. All reported p-values were two-sided, and statistical significance was set at P ≤ 0.05.

## 3 Results

### 3.1 Included studies

Using a predefined search strategy (**Table 1**), we systematically searched the PubMed, Cochrane Library, and EMBASE databases, along with major conference websites (ASCO, ESMO, SABCS), identifying a total of 1312 related articles. After screening the titles and abstracts, we selected 9 articles. We carefully reviewed the full texts, excluded 4 of them (**Supplementary Table 1**), and ultimately included 5 studies (18–22). The study selection process is shown in **Figure 1**.

**Table 1:** Literature search strategy.

#1	Phosphatidylinositol 3 Kinases OR phosphoinositide 3 kinase inhibitors OR phosphatidylinositol 3 kinase inhibitors OR PI3K inhibitors OR Dactolisib OR Idelalisib OR Buparlisib OR Alpelisib OR Voxtalisib OR Omipalisib OR Apitolisib OR Duvelisib OR Gedatolisib OR Copanlisib OR Pilaralisib OR Taselisib OR LY 3023414 OR Pictilisib OR Zydelig OR Piqray OR Aliqopa OR Serabelisib OR Umbralisib OR AZD8186
#2	Breast Neoplasms OR breast cancer OR breast tumor OR breast tumour OR breast neoplasm OR mammary cancer OR mammary carcinoma OR breast carcinoma OR breast cancers OR breast tumors OR breast tumours OR breast neoplasms OR mammary cancers OR mammary carcinomas OR breast carcinomas
#3	fulvestrant
#4	clinical trial OR randomized controlled trial OR randomised controlled trial OR randomized controlled OR randomised controlled OR RCT
#5	#1 AND #2 AND #3 AND #4

**Figure 1:**
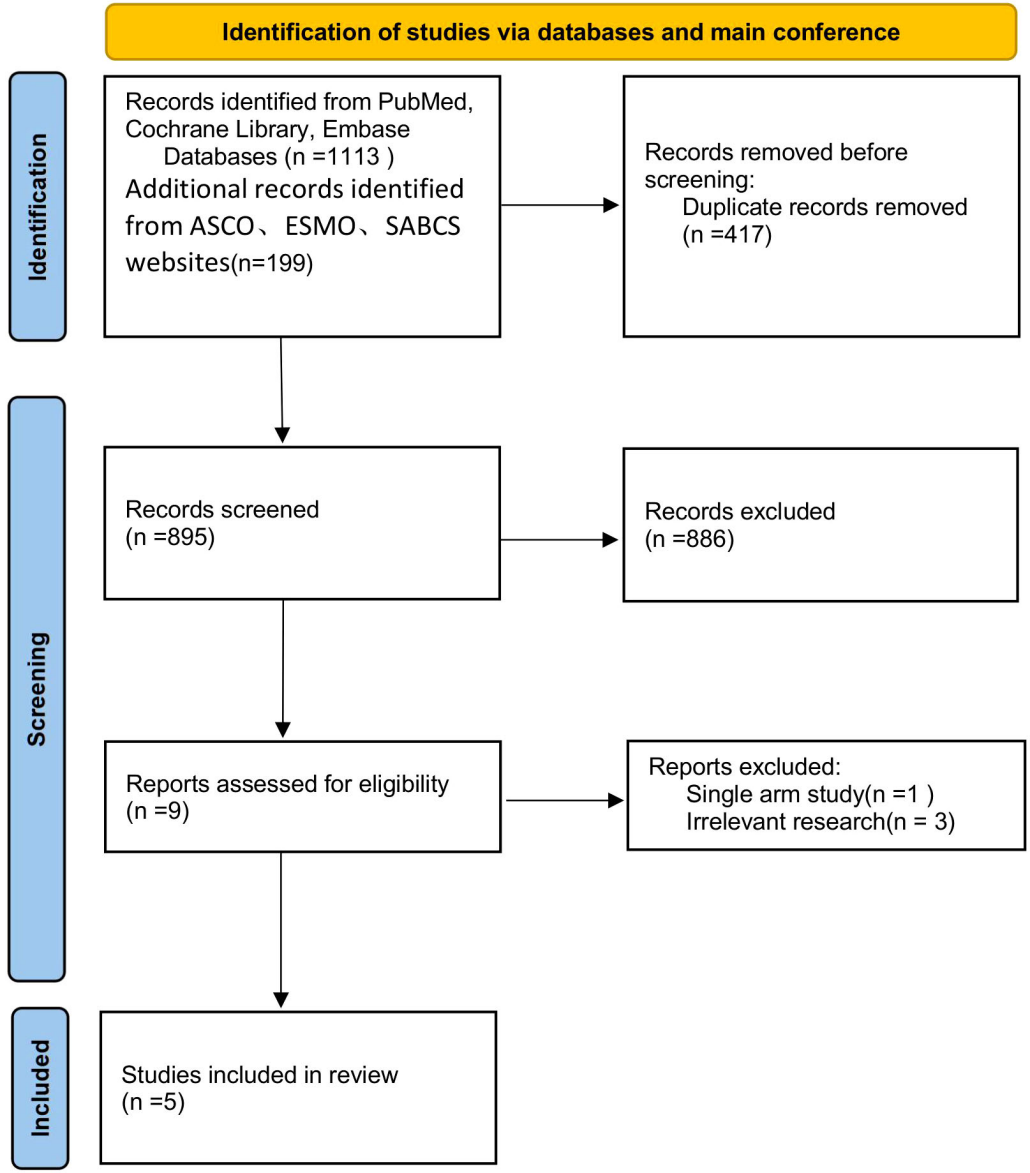
Flow chart for the identification of included studies.

### 3.2 Baseline characteristics of included studies

All included trials were randomized controlled clinical studies, comprising one Phase II and four Phase III studies. The detailed characteristics of these trials are summarized in **Table 1**. For the SOLAR-1 and SANDPIPER trials, which did not report complete PFS data, we derived the results by synthesizing PFS data from patients with and without PIK3CA mutations confirmed through tumor tissue testing. The FERGI trial consisted of two parts, from which we extracted and integrated relevant outcomes into our analysis. Regarding treatment interventions, three studies investigated pan-PI3K inhibitors, while two evaluated specific PI3K inhibitors (**Table 2**).

**Table 2:** Characteristics of included studies.

Trial	Author	Year	Phase	Patients	Visceral disease(n/Total)		Cancer stage	Liver metastasis(n/Total)		Median age	Types of PI3K inhibitors	Intervention measures		Primary endpoint
					T	C		T	C			T	C	
SOLAR-1	André	2019	III	HR(+)/HER2(-)	159/284	174/288	III/IV	90/284	90/288	63	specific PI3K inhibitors	Alpelisib + Fulvestrant	Placebo + Fulvestrant	PFS
BELLE-2	Baselga	2017	III	HR(+)/HER2(-)	341/576	337/571	III/IV	/	/	61	pan-PI3K inhibitors	Buparlisib + Fulvestrant	Placebo + Fulvestrant	PFS
BELLE-3	Di Leo	2018	III	HR(+)/HER2(-)	212/289	103/143	III/IV	137/289	76/143	61	pan-PI3K inhibitors	Buparlisib + Fulvestrant	Placebo + Fulvestrant	PFS
SANDPIPER	Dent	2021	III	HR(+)/HER2(-)	254/417	129/214	III/IV	/	/	60	specific PI3K inhibitors	Taselisib + Fulvestrant	Placebo + Fulvestrant	PFS
FERGI	Krop	2016	II	HR(+)/HER2(-)	72/130	52/99	III/IV	/	/	62	pan-PI3K inhibitors	Pictilisib + Fulvestrant	Placebo + Fulvestrant	PFS

### 3.3 Progression-free survival

**Figure 2:**
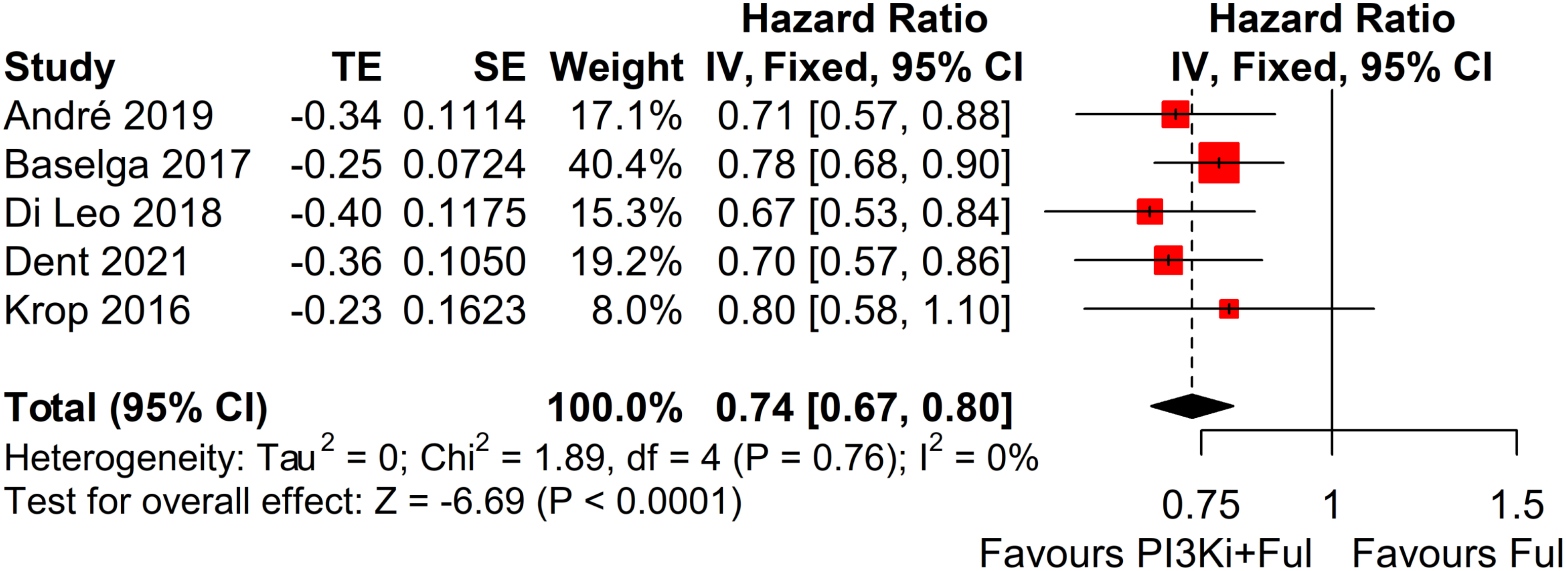
Forest plot for progression-free survival (PFS). CI, confidence interval; HR, hazard ratio; PI3Ki, PI3K inhibitors; Ful, fulvestrant.

This systematic review and meta-analysis included five randomized controlled trials (RCTs), encompassing a total of 3011 patients with advanced breast cancer. All studies reported PFS outcomes. For the SOLAR-1 and SANDPIPER trials, which did not report PFS for the overall population, we estimated PFS by integrating data from patients with and without PIK3CA mutations identified in tumor tissue. The results showed that, compared with fulvestrant alone, PI3K inhibitors in combination with fulvestrant significantly improved PFS (HR = 0.74, 95% CI: 0.67–0.80, P < 0.0001). The pooled analysis revealed statistically significant results with minimal heterogeneity across the studies (I² = 0%, P = 0.76) (**Figure 2**).

### 3.4 Objective response rate and clinical benefit rate analysis

**Figure 3:**
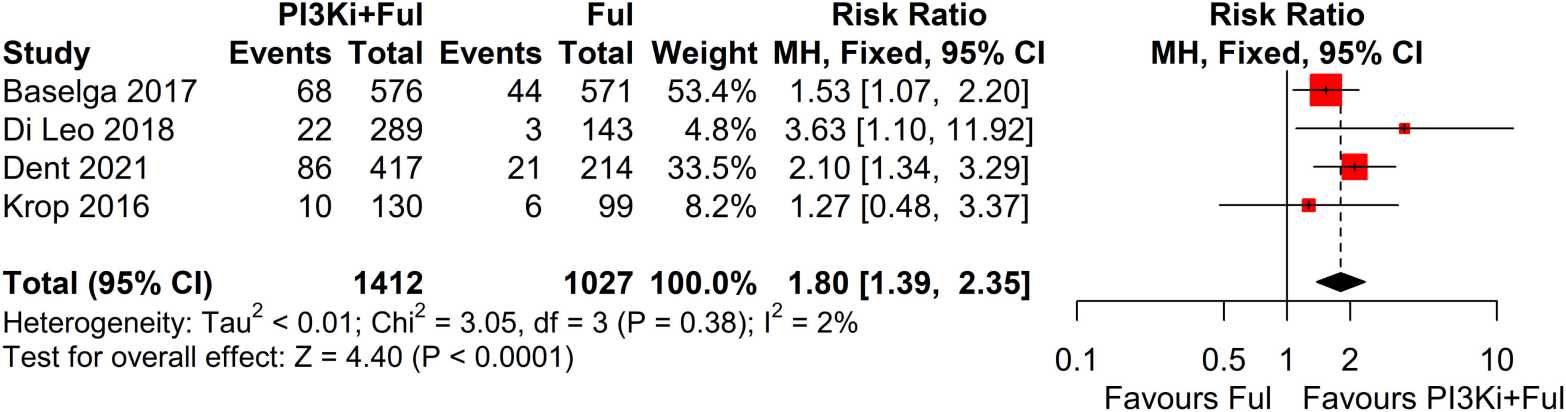
Forest plot for overall response rate (ORR).

**Figure 4:**
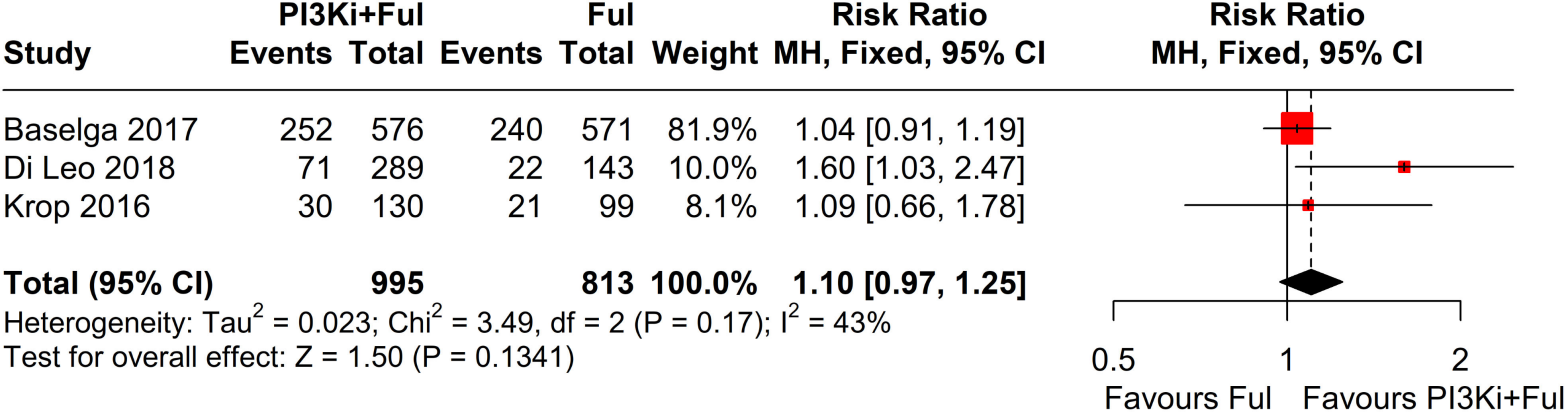
Forest plot for clinical benefit rate (CBR).

Four studies reported complete data on ORR. Since heterogeneity among studies was relatively low (I² = 2%, P = 0.38), a fixed-effects model was used. The results showed that PI3K inhibitors combined with fulvestrant significantly increased ORR (RR = 1.80, 95% CI: 1.39–2.35, P<0.0001) (**Figure 3**). However, there was no statistically significant difference in clinical benefit rate (CBR) between the two groups (RR=1.10, 95% CI: 0.97–1.25, P=0.1341) (**Figure 4**).

### 3.5 Safety

**Figure 5:**
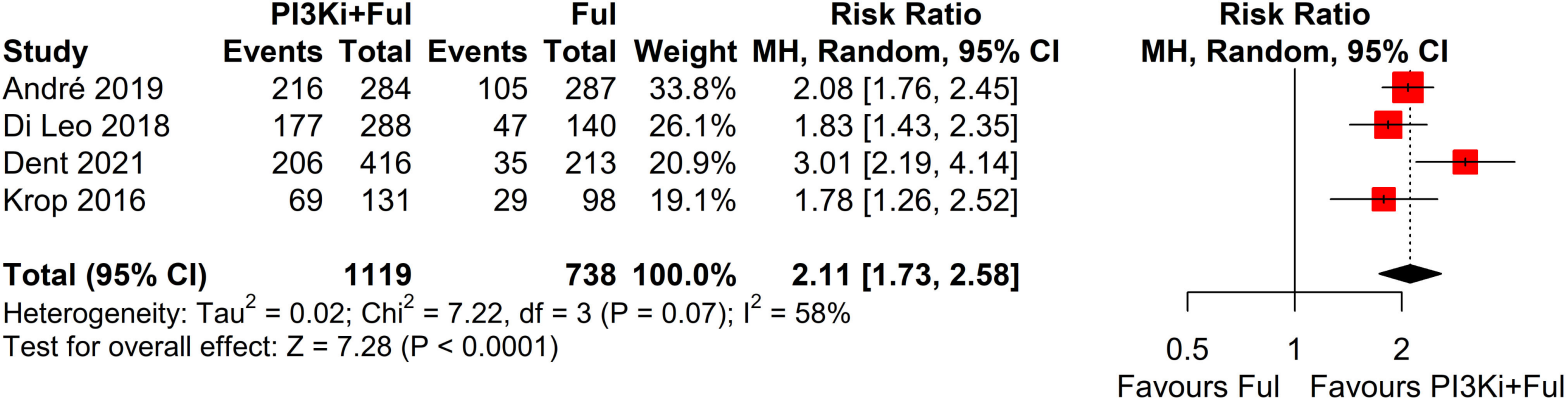
Forest plot for grade ≥3 adverse events.

**Figure 6:**
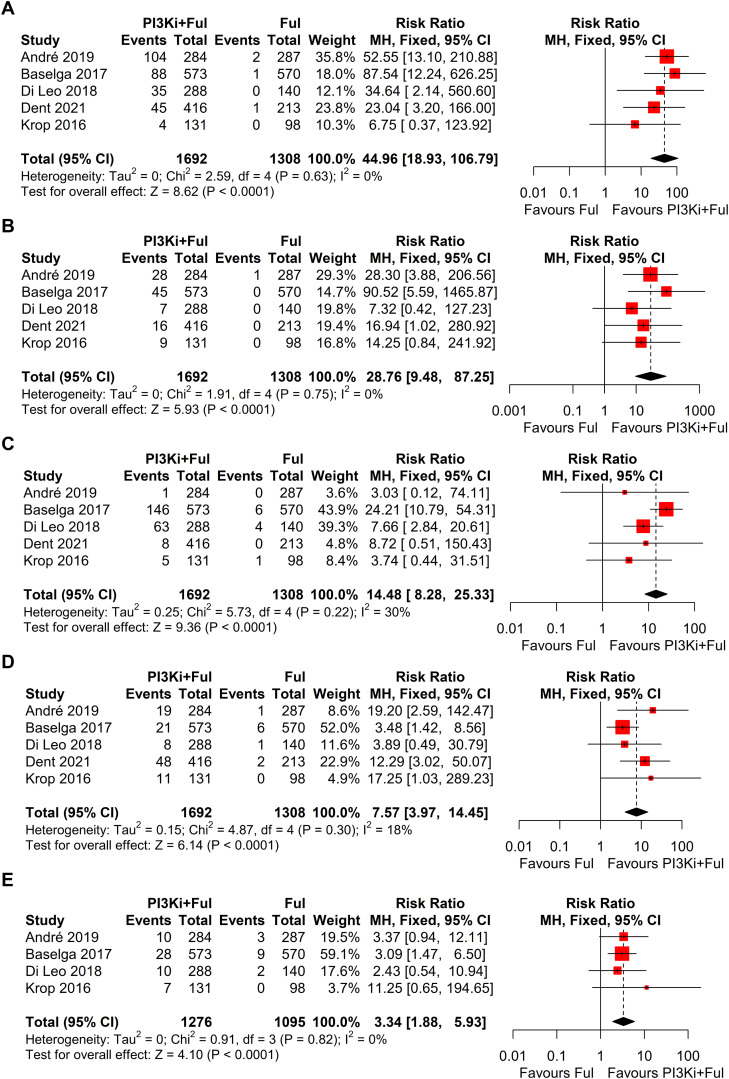
Forest plots for top five grade ≥3 adverse events: **(A)**, Hyperglycemia; **(B)**, Rash; **(C)**, Transaminitis (ALT); **(D)**, Fatigue; **(E)**, Diarrhoea.

**Figure 7:**
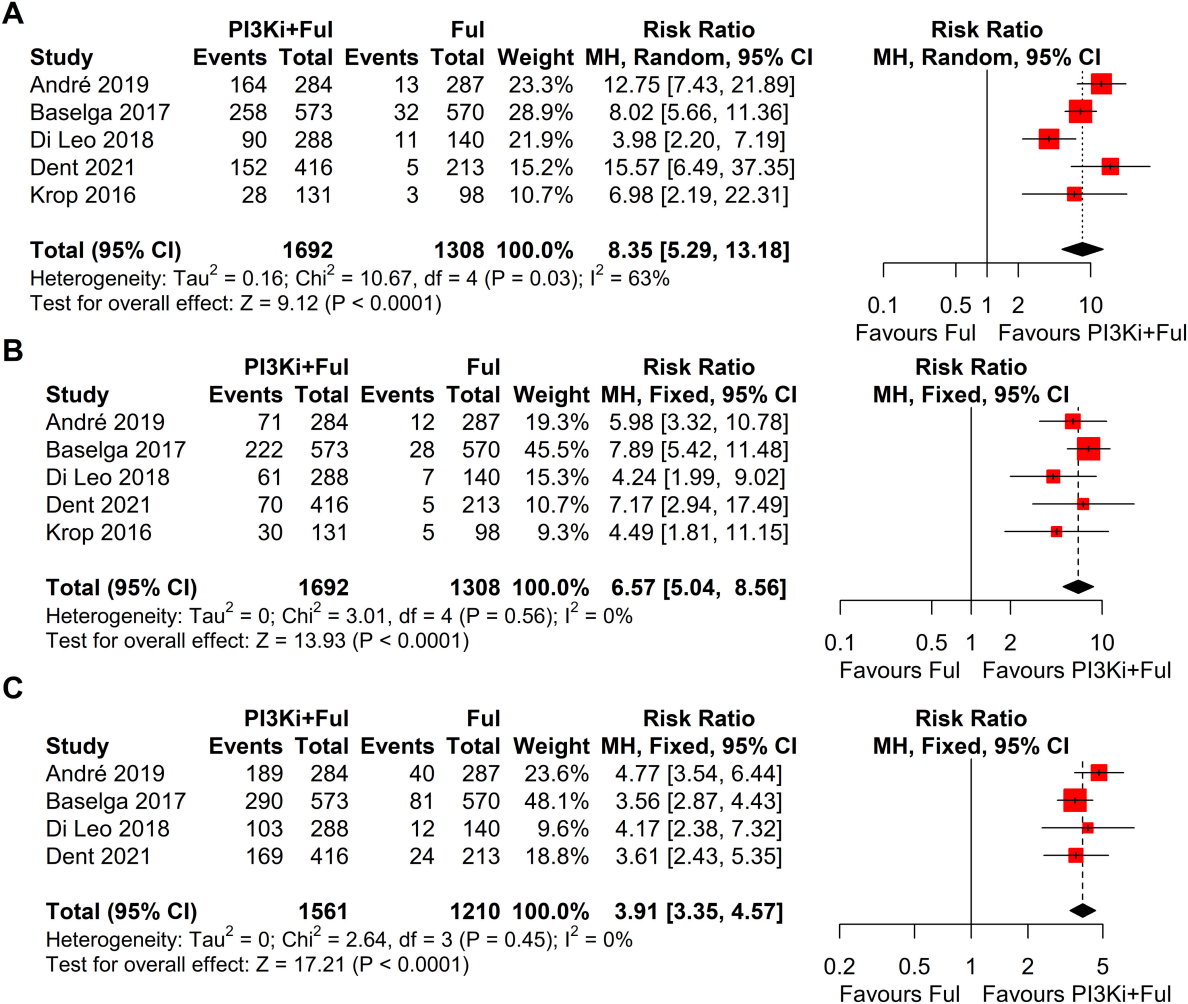
Forest plots of dose adjustment events for pi3k inhibitors vs. placebo: **(A)**, Dose reductions; **(B)**, Dose interruptions; **(C)**, Dose discontinuations.

Based on safety data from five randomized controlled trials (RCTs), the incidence of grade ≥3 adverse events was significantly higher in patients receiving PI3K inhibitors combined with fulvestrant (RR = 2.11, 95% CI: 1.73–2.58, P < 0.0001), with significant heterogeneity observed (I² = 58%) (**Figure 5**). We conducted a sensitivity analysis using the leave-one-out method and found that when the SANDPIPER study was removed, I² decreased to 0, but the effect size remained significant (RR = 1.97, 95% CI: 1.73–2.24) (**Supplementary Figure 1**). Meta-regression analysis results showed that the publication year of studies (β = 0.1075, 95% CI: 0.0178, 0.1972, p = 0.0188) significantly influenced the treatment effect. The regression model’s R² was 100.00%, indicating that the model explained 100% of the heterogeneity. The heterogeneity of the regression model was low, with I² = 0.00% (p = 0.4786), suggesting no significant heterogeneity between studies (**Supplementary Figure 2**). The top five adverse events showed a significant increase, particularly hyperglycemia, rash, and transaminitis (ALT) (**Figure 6**). In addition, combination therapy significantly increased the risks of dose reductions, dose interruptions, and dose discontinuations (**Figure 7**).

### 3.6 Subgroup analysis

**Figure 8:**
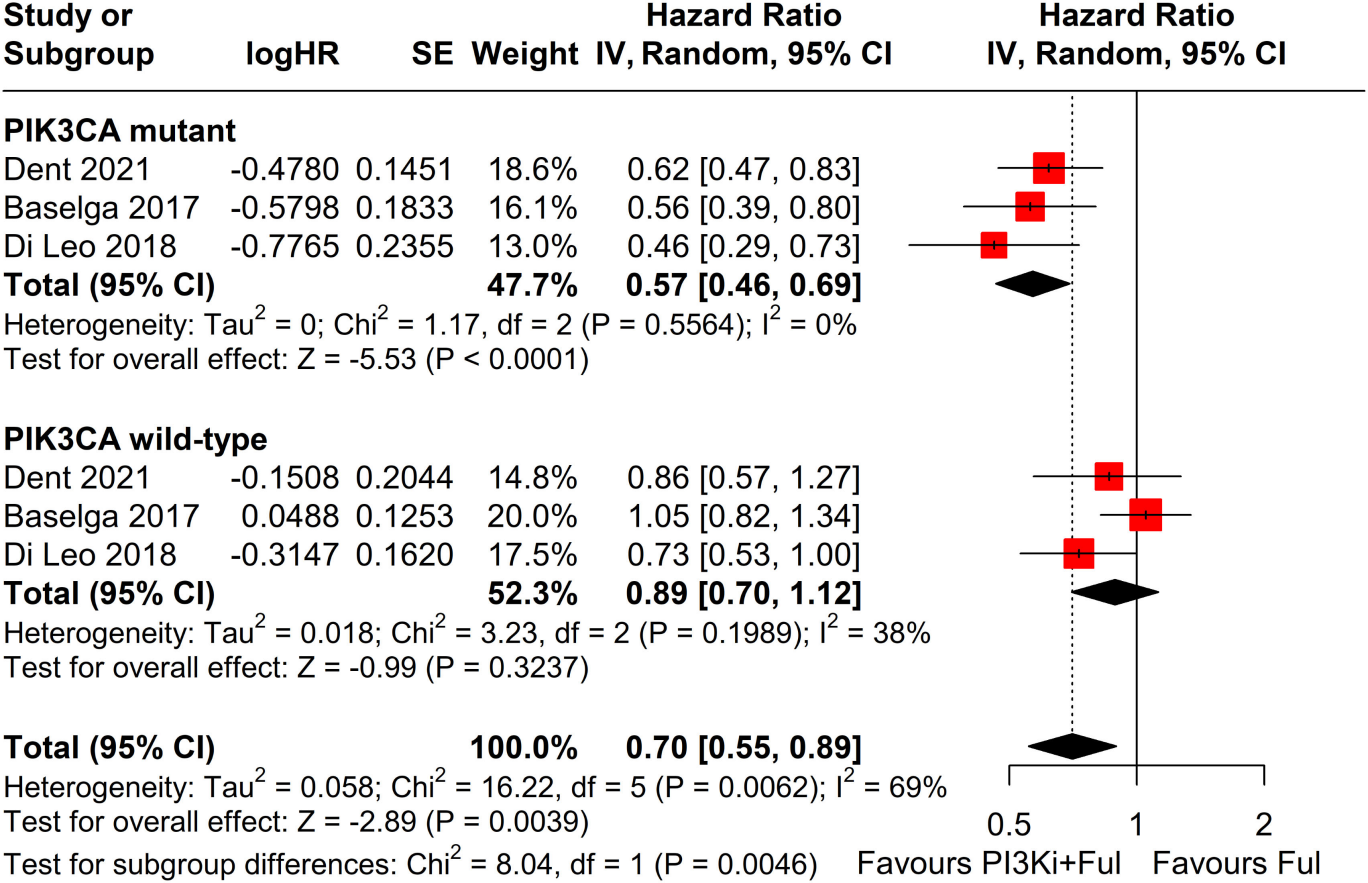
Subgroup analysis of progression-free survival (PFS) stratified by PIK3CA mutation status confirmed by ctDNA analysis.

**Figure 9:**
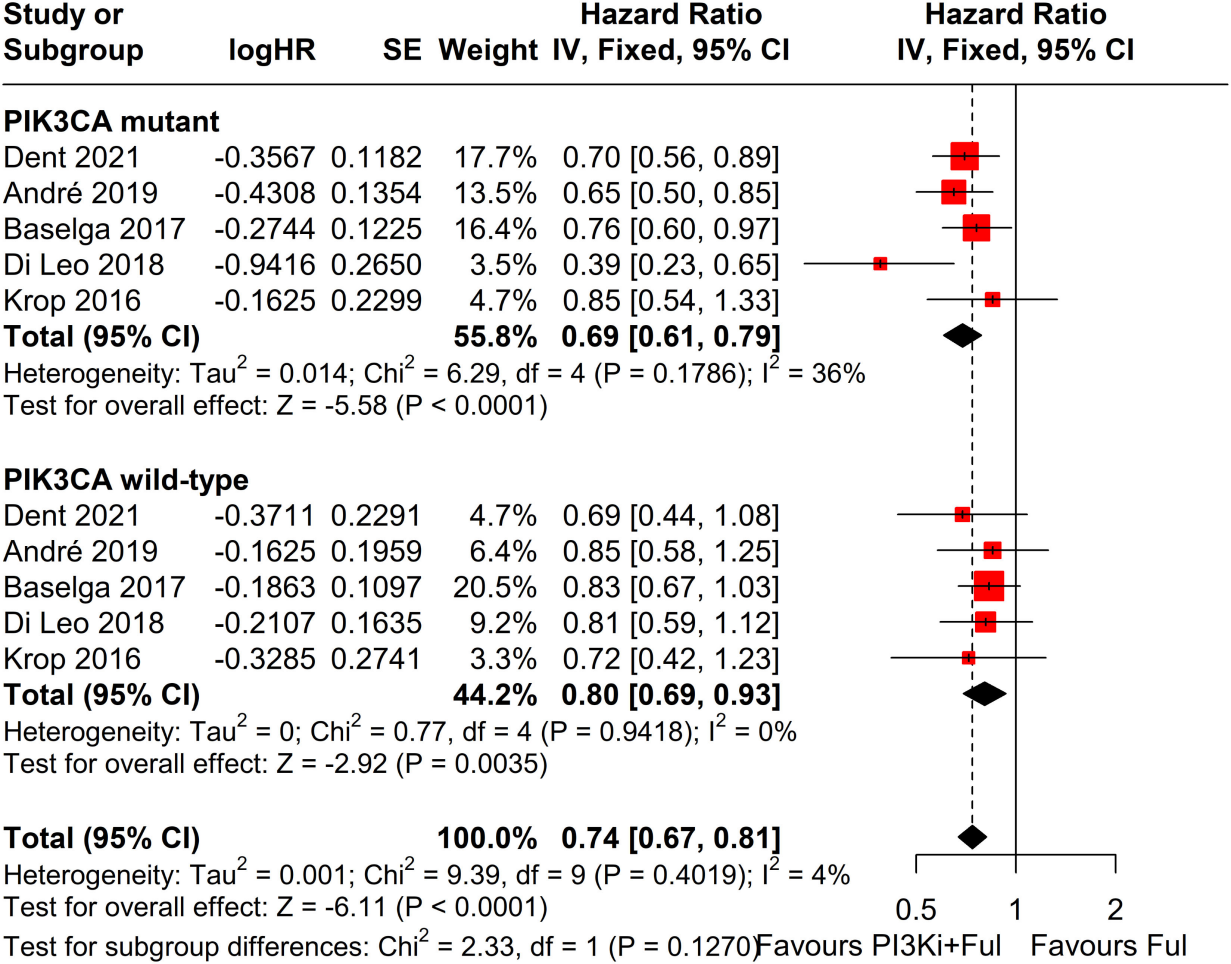
Subgroup analysis of progression-free survival (PFS) stratified by PIK3CA mutation status confirmed by tumor tissue.

**Figure 10:**
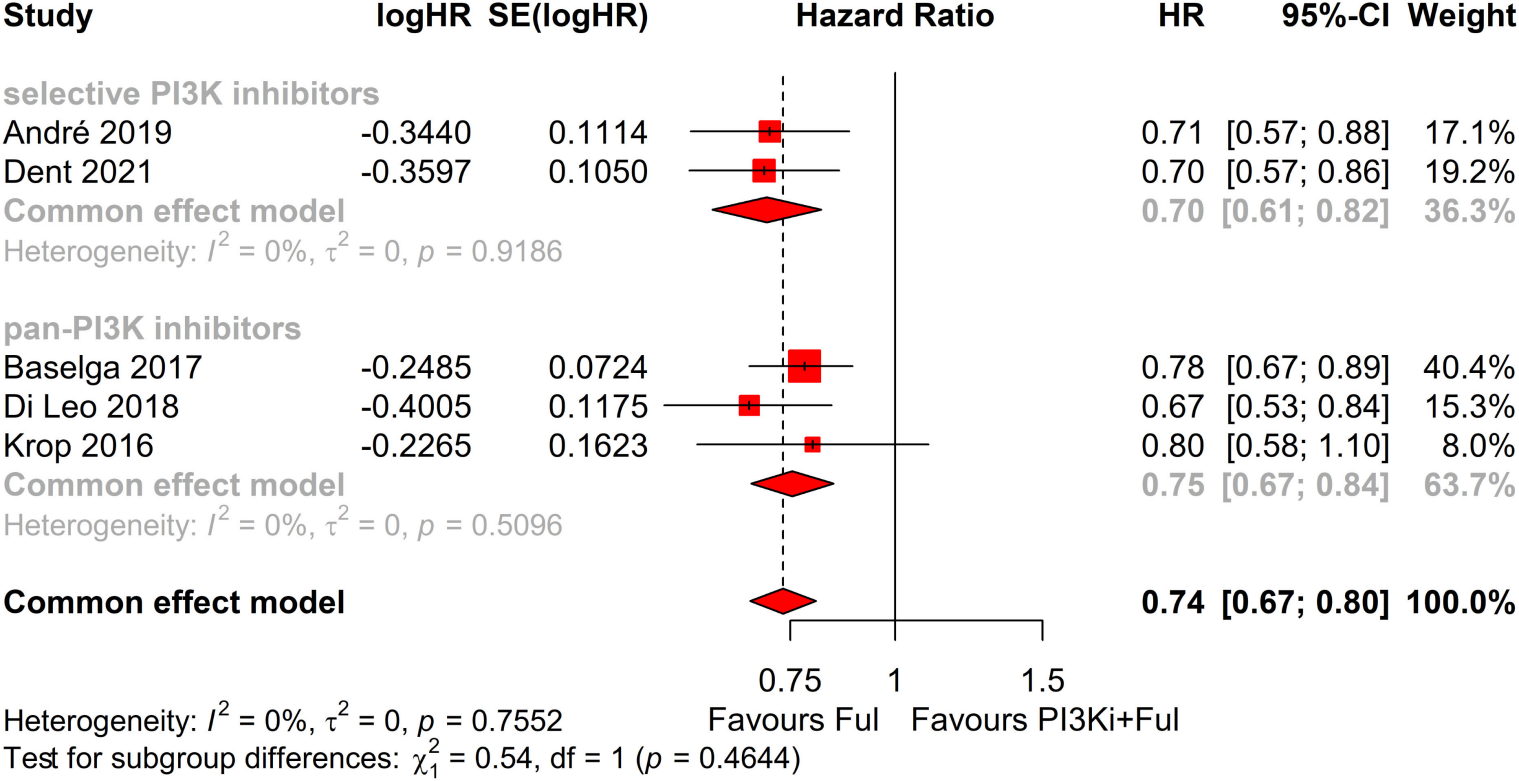
Subgroup analysis of progression-free survival (PFS) based on different types of PI3K inhibitors: selective vs pan-PI3K Inhibitors.

**Figure 11:**
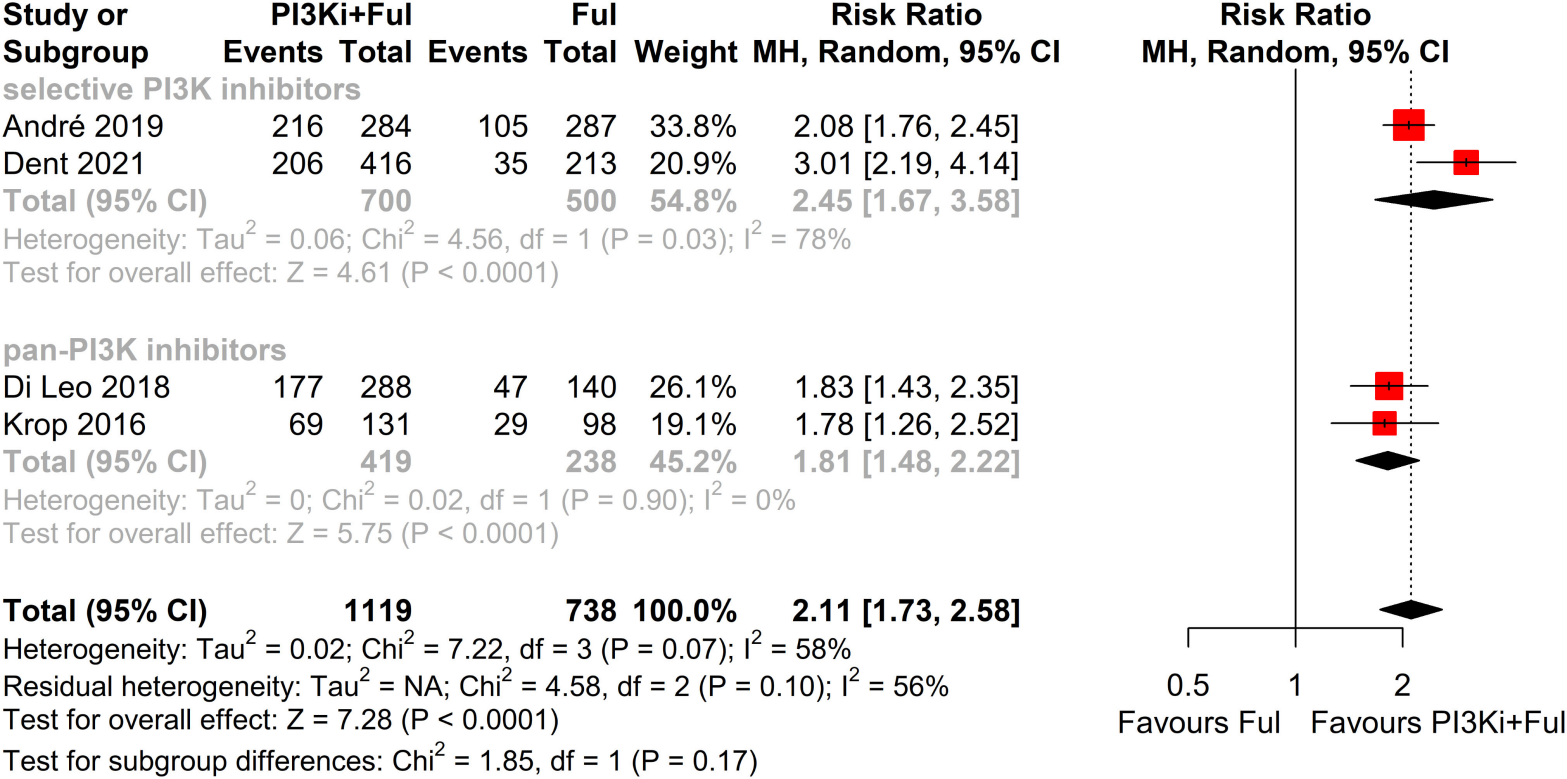
Subgroup analysis of grade ≥3 adverse events stratified by PIK3CA mutation status confirmed by tumor tissue.

Subgroup analysis revealed that patients with PIK3CA mutations identified through ctDNA testing demonstrated a significantly improved PFS compared to those with the wild-type PIK3CA(P=0.0039) (**Figure 8**). Due to high heterogeneity among studies (I²=69%, P=0.0062), a random-effects model was applied. In contrast, no statistically significant difference in PFS was observed between groups of patients with PIK3CA mutation status detected on tumor tissue testing (P=0.1270), with low heterogeneity (I²=4%, P=0.4019) (**Figure 9**). Further analysis of the effects of different types of PI3K inhibitors on PFS revealed no significant difference between pan-PI3K inhibitors and specific PI3K inhibitors (P=0.4644), with low heterogeneity (I²=0%, P=0.7552) (**Figure 10**). Additionally, a comparison of the adverse event incidence rates between the two types of PI3K inhibitors showed no statistically significant difference (P=0.17). However, heterogeneity was high (I² = 58%, P = 0.07), necessitating the use of a random-effects model (**Figure 11**). We also calculated the treatment discontinuation rate and interruption rate for pan-PI3K inhibitors and specific PI3K inhibitors. The results were 45.64% *vs*. 51.14% and 31.55% *vs*. 20.14%, respectively.

### 3.7 Risk of bias

**Figure 12:**
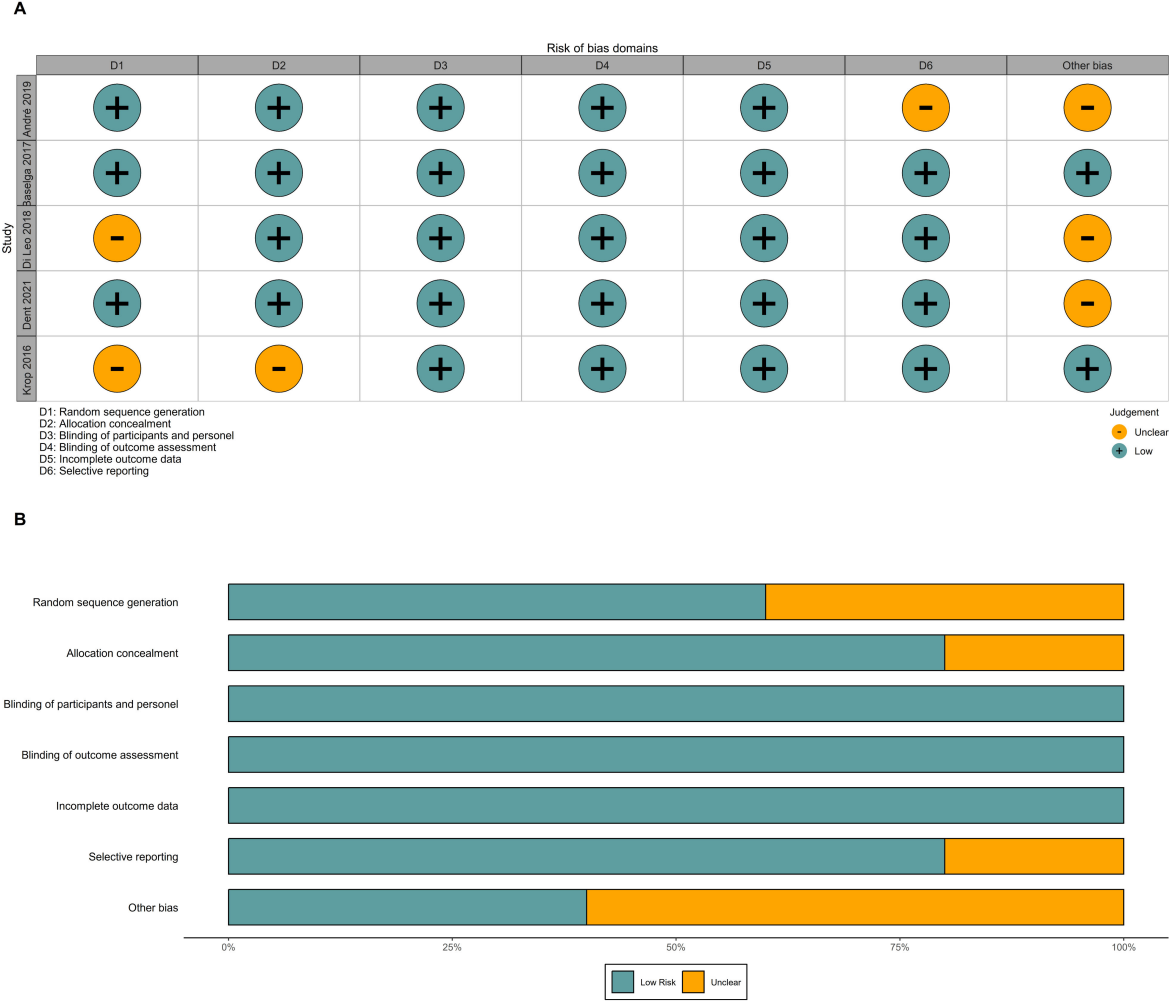
Risk of bias. **(A)** Risk of bias graph of included RCTs. **(B)** Risk of bias summary of included RCTs. RCTs, randomized controlled trials.

Overall, the risk of bias in this study was relatively low, as all included studies were well-designed randomized, double-blind trials. However, two studies did not clearly describe their methods of random sequence generation and allocation concealment. In the FERGI trial, the dose of pictilisib was reduced due to toxicity concerns, while the SOLAR-1 trial did not report PFS in patients with PIK3CA mutations identified by ctDNA (**Figure 12**).

## 4 Discussion

The PI3K/AKT/mTOR (PAM) signaling pathway plays a key role in cellular physiological functions, extensively participating in critical processes such as cell growth, proliferation, and signal transduction (10, 11). Studies have shown that in hormone receptor–positive (HR+) breast cancer, PIK3CA mutations activate the PI3K pathway, promoting ligand-independent estrogen receptor (ER) activation, thereby leading to endocrine therapy resistance and accelerating disease progression (23, 24). Consequently, targeting key nodes within the PI3K/AKT/mTOR (PAM) pathway represents a promising therapeutic strategy to improve clinical outcomes in patients with advanced breast cancer.

This systematic review and meta-analysis included five randomized controlled trials and demonstrated that combining PI3K inhibitors with fulvestrant confers significant clinical benefits in advanced breast cancer, particularly by prolonging PFS and enhancing the ORR. Compared with fulvestrant alone, adding PI3K inhibitors reduced the risk of disease progression by 26% and increased the ORR by 80%, thereby significantly improving overall patient prognosis. These findings highlight the potential value of PI3K inhibitors plus fulvestrant in the therapeutic landscape of advanced breast cancer. Although the clinical benefit rate (CBR) in the PI3K inhibitor plus fulvestrant treatment group did not differ significantly from that in the control group (P=0.1341), the small sample size in the included studies increased the risk of a type II error. Future research with larger sample sizes is needed to provide more robust evidence to support this conclusion.

Despite the remarkable efficacy of PI3K inhibitors combined with fulvestrant in advanced breast cancer, the incidence of grade ≥3 adverse events was significantly higher in patients receiving PI3K inhibitors combined with fulvestrant (RR = 2.11), with high heterogeneity observed (I² = 58%). We conducted a sensitivity analysis using the leave-one-out method and found that when the SANDPIPER study was removed, I² decreased to 0, while the effect size remained significant (RR = 1.97). Further meta-regression analysis showed that the publication year of studies explained 100% of the heterogeneity (p < 0.05). We attempted to analyze patient demographics, dosing schedules, and other influencing factors, but no obvious abnormalities were found. Based on the sensitivity analysis results, the overall conclusions were not affected. The top five adverse events showed a significant increase, particularly hyperglycemia (RR = 44.96). This observation may be related to the mechanism of action of PI3K inhibitors. The PI3K/AKT/mTOR signaling pathway is widely involved in insulin signal transduction and is closely related to glucose metabolism. When exogenous insulin binds to its receptor, the receptor is activated, triggering downstream signaling through the PAM pathway to facilitate glucose transport. Consequently, inhibiting this pathway impairs cellular glucose uptake, thereby elevating blood glucose levels (25–27). In clinical practice, advanced breast cancer patients with concomitant diabetes should receive enhanced blood glucose monitoring and preventive interventions to reduce the risks associated with hyperglycemia (28–30). Additionally, rash and transaminitis (ALT) are also important adverse events that require attention during PI3K inhibitor therapy. The frequent occurrence of these adverse events significantly increases the risks of dose reductions, dose interruptions, and treatment discontinuations (RR = 8.70, 6.57, and 3.91, respectively). Therefore, close monitoring and timely intervention for these adverse events are critical for improving patients’ treatment tolerance. Since most patients in the included studies received subsequent lines of therapy following disease progression, none of the studies reported complete overall survival (OS) data. Consequently, this meta-analysis did not assess OS.

Subgroup analysis demonstrated that patients with PIK3CA mutations detected through ctDNA achieved a significantly greater PFS benefit compared to those with the wild-type variant, highlighting the pivotal role of mutation status in predicting treatment efficacy. Detecting PIK3CA mutations via ctDNA may offer a valuable biomarker for evaluating therapeutic efficacy and guiding precision medicine strategies. However, the pre-specified subgroup analysis based on PIK3CA mutation status identified via tumor tissue did not demonstrate a statistically significant difference in PFS (p = 0.1514). The inconsistency between ctDNA and tumor tissue detection results may be attributed to several factors. One key reason is the spatial heterogeneity of PIK3CA mutations within tumor tissues. Due to sampling limitations, archived tissue samples may fail to capture the full mutational landscape of the tumor. In contrast, ctDNA analysis reflects a broader genetic profile, as it captures DNA fragments shed from tumor cells throughout the body, thereby increasing the likelihood of detecting specific mutations (31). Furthermore, the mutation status of PIK3CA may evolve over time and in response to treatment. Circulating tumor DNA (ctDNA) offers a dynamic reflection of these changes, while archived tumor tissue may not accurately represent the current mutation status (32, 33). Additionally, the sensitivity of ctDNA detection is influenced by factors such as tumor size, location, and pathological characteristics, which may limit the detection of smaller tumors or low-frequency mutations. In contrast, tissue samples often contain a higher concentration of tumor DNA, which makes it easier to identify mutations (34).

Conventional perspectives suggested that selective PI3K inhibitors minimized interference with normal physiological processes by specifically targeting the class IA catalytic subunit of PI3K(p110α), thereby lowering the incidence of adverse events. In contrast, pan-PI3K inhibitors block the kinase activity of all four isoforms of class I PI3K (α, β, δ, and γ) and exert broad effects on various physiological processes through downstream signaling molecules, which inevitably increases drug-related toxicity (35–37). However, our subgroup analysis indicated that the incidence of grade ≥3 adverse events was significantly higher with selective PI3K inhibitors compared to pan-PI3K inhibitors. This discrepancy may be attributed to the duration of drug exposure. Due to poor tolerability, pan-PI3K inhibitors are more likely to lead to dose reductions, dose interruptions, and treatment discontinuations, ultimately shortening the overall exposure time. To verify the hypothesis, we further analyzed the treatment discontinuation rate and interruption rate for pan-PI3K inhibitors and selective PI3K inhibitors. The results showed that the interruption rate of pan-PI3K inhibitors was significantly higher than that of selective PI3K inhibitors. We also noted that there was considerable heterogeneity in the subgroup analysis. Due to the limited number of studies included, we were unable to conduct further analysis, and this issue needs to be explored in future research.

### 4.1 Limitations and future research directions

This systematic review and meta-analysis still have several limitations. First, the number of randomized controlled trials included is relatively small, comprising only five studies with a total of 3,011 patients. This limited sample size may affect the statistical power of the results, particularly in detecting small differences, thus increasing the risk of Type II errors. Future research should aim to enhance statistical power by increasing the sample size. In addition, the use of aggregate data instead of individual patient data reduces the reliability of the study’s conclusions. Furthermore, variations in patient baseline characteristics, study designs, and treatment regimens across the included studies may introduce heterogeneity, thereby affecting the reliability of the results. Notably, three of the included studies used pan-PI3K inhibitors, whereas two adopted selective PI3K inhibitors, potentially increasing inter-study heterogeneity. Thus, additional rigorously conducted and standardized studies are imperative to validate and further refine these findings.

More selective PI3K inhibitors remain under active development. For instance, results from the INAVO120 trial suggest that the selective PI3K inhibitor inavolisib, when administered in combination with palbociclib and fulvestrant, can significantly improve PFS (38). Compared with palbociclib plus fulvestrant alone, this combination regimen extended PFS to 15.0 months (versus 7.3 months), with a hazard ratio of 0.43 (95% CI: 0.32–0.59, p < 0.001). Because the study did not meet the inclusion criteria, it was excluded from the current meta-analysis. Furthermore, another Phase III trial (INAVO121) is evaluating inavolisib plus fulvestrant in comparison to alpelisib plus fulvestrant. The results of this trial are anticipated to further clarify the potential value of selective PI3K inhibitors in advanced breast cancer treatment.

### 4.2 Innovation and clinical significance

This study provided the first systematic review and meta-analysis of the efficacy and safety of combining PI3K inhibitors with fulvestrant in the treatment of advanced breast cancer, revealing the potential value of this combination strategy. Through subgroup analysis, we further elucidated the relationship between PIK3CA mutation status and treatment response, and proposed that ctDNA-based detection of PIK3CA mutations could serve as a potential biomarker for predicting treatment outcomes. This finding provided new perspectives for individualized therapy and helped optimize treatment strategies.

### 5 Conclusion

The combination of PI3K inhibitors and fulvestrant significantly improved PFS and ORR in patients with advanced breast cancer. However, this treatment regimen was associated with a notable increase in adverse events, particularly hyperglycemia, rash, and transaminitis (ALT). In patients with PIK3CA mutations detected on ctDNA analysis, PFS was significantly improved compared to those with wild-type PIK3CA, suggesting that ctDNA-based PIK3CA mutation status may serve as a potential biomarker for treatment response.

## Data Availability

The original contributions presented in the study are included in the article/**Supplementary Material**. Further inquiries can be directed to the corresponding author.

## Supplementary material

Supplementary Figure 1 Sensitivity analysis of grade ≥3 adverse events using leave-one-out method.

Supplementary Figure 2 Bubble plot of meta-regression analysis for grade ≥3 adverse events by publication year.

Supplementary Table 1 Details of 4 excluded studies after full-text article screening.
