# Supplementary figures and images for: Efficacy and safety of PI3K inhibitors combined with fulvestrant for HR+/HER2− advanced breast cancer: a systematic review and meta-analysis
Source: Front Oncol. 2025 Jun 4;15:1556978. doi: 10.3389/fonc.2025.1556978 (PMC12174159; doi:10.3389/fonc.2025.1556978)

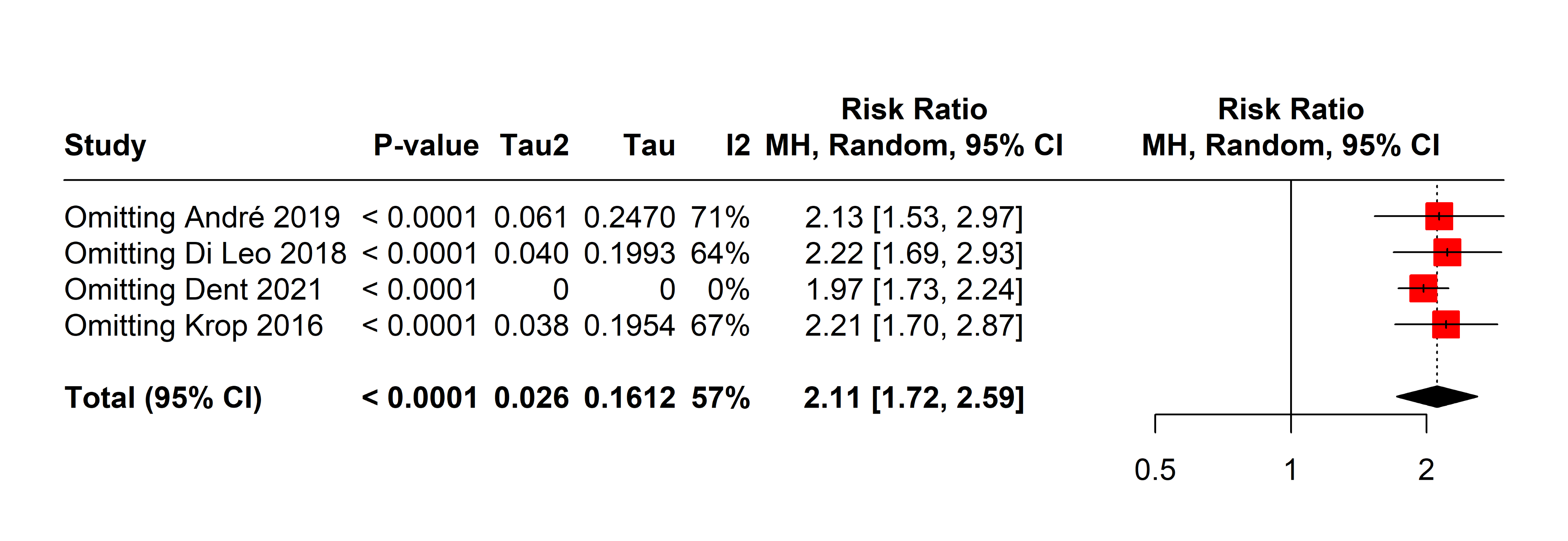

Supplement: Supplementary Figure 1 — Sensitivity analysis of grade ≥3 adverse events using leave-one-out method. [file Image1.tif]

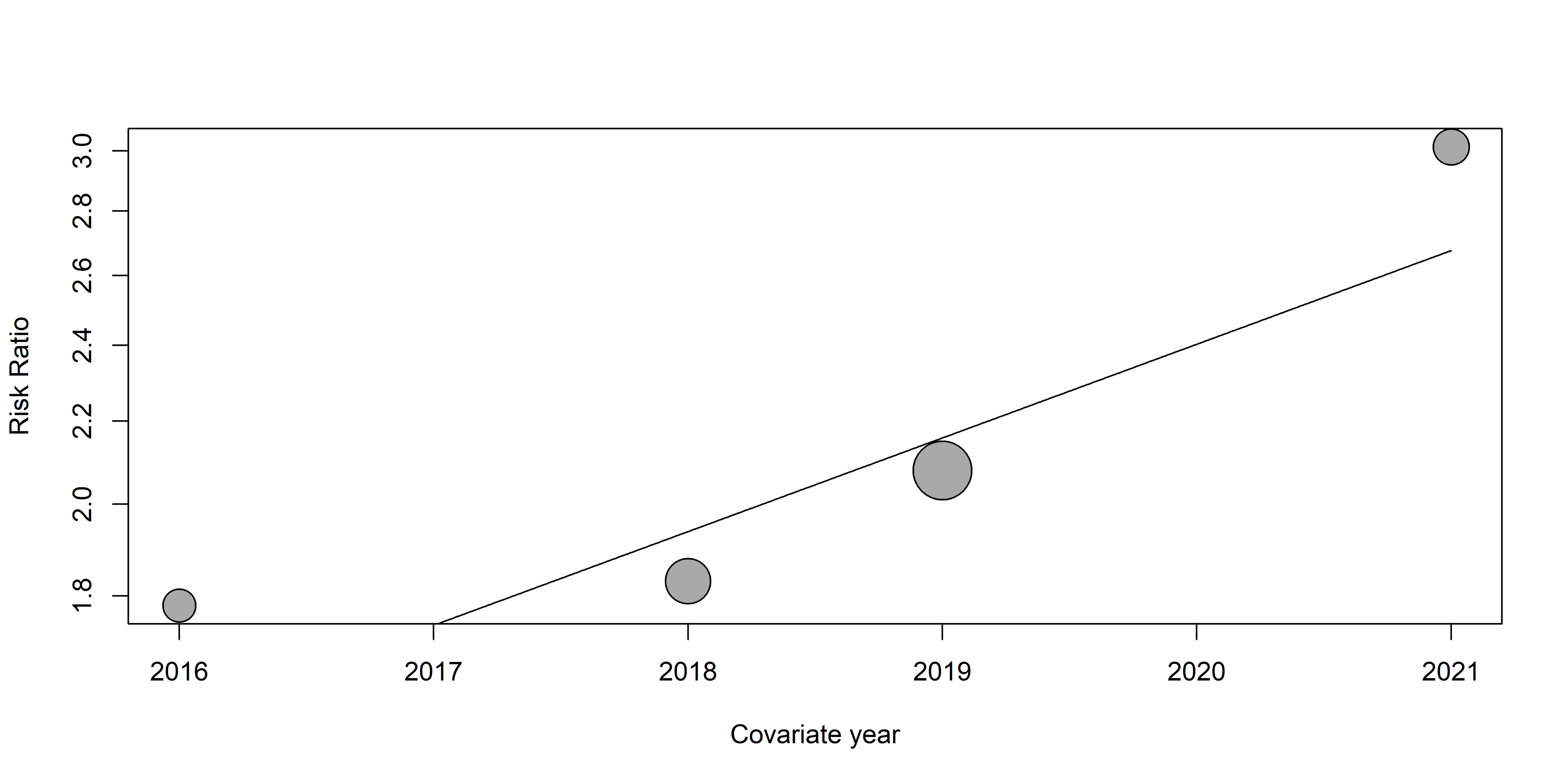

Supplement: Supplementary Figure 2 — Bubble plot of meta-regression analysis for grade ≥3 adverse events by publication year. [file Image2.tif]
