# Supplementary material for: Efficacy and safety of PI3K inhibitors combined with fulvestrant for HR+/HER2− advanced breast cancer: a systematic review and meta-analysis
Source: Front Oncol. 2025 Jun 4;15:1556978. doi: 10.3389/fonc.2025.1556978 (PMC12174159; doi:10.3389/fonc.2025.1556978)
Supplement: Supplementary Table 1 — Details of 4 excluded studies after full-text article screening. [file Table1.docx]

| **Trial name** | **Author** | **Year** | **Phase** | **Exclusion reason** | **DOI** |
| --- | --- | --- | --- | --- | --- |
| BELLE-4 | M Martín | 2017 | Ⅱ | The intervention includes Paclitaxel, which does not meet the inclusion criteria. | DOI: 10.1093/annonc/mdw562 |
| BYLieve | Hope S | 2021 | Ⅱ | This is a single-arm study, which does not meet our inclusion criteria. | DOI: 10.1016/S1470-2045(21)00034-6 |
| INAVO120 | Nicholas C. | 2024 | Ⅲ | The intervention in the experimental group includes CDK4/6 inhibitors, which does not meet our inclusion criteria. | DOI: 10.1056/nejmoa2404625 |
| NEO-ORB | Ingrid A | 2019 | Ⅱ | The study involves neoadjuvant therapy, and the included patients are those eligible for surgery, which does not meet the exclusion criteria. | DOI: 10.1158/1078-0432.CCR-18-3160 |
